# Association between dietary inflammatory index and gallstones in US adults

**DOI:** 10.3389/fnut.2024.1403438

**Published:** 2024-05-03

**Authors:** Yanling Luo, Xiaolian Gao, Mingzhong Xiao, Fen Yang, Xinhong Zhu, Guiyuan Qiao, Cong Xiang, Junxiu Tao

**Affiliations:** ^1^School of Nursing, Hubei University of Chinese Medicine, Wuhan, China; ^2^Hubei Shizhen Laboratory, Wuhan, China; ^3^Department of Hepatology, Hubei Key Laboratory of The Theory and Application Research of Liver and Kidney in Traditional Chinese Medicine, Hubei Provincial Hospital of Traditional Chinese Medicine, Wuhan, China; ^4^Affiliated Hospital of Hubei University of Chinese Medicine, Wuhan, China; ^5^Hubei Province Academy of Traditional Chinese Medicine, Wuhan, China

**Keywords:** dietary inflammatory index, gallstone, propensity score matching, restrictive cubic spline, NHANES

## Abstract

**Introduction:**

Previous studies have found that diet’s inflammatory potential is related to various diseases. However, little is known about its relationship with gallstones. The present study aims to investigate the relationship between dietary inflammatory index (DII) and gallstones.

**Methods:**

Data were obtained from the 2003–2020 National Health and Nutrition Examination Survey (NHANES). We used the nearest neighbor propensity score matching (PSM) with a ratio of 1:1 to reduce selection bias. Logistic regression models estimated the association between DII and gallstones. The non-linear relationship was explored with restricted cubic splines (RCS). BMI subgroup stratification was performed to explore further the connection between DII and gallstones in different populations.

**Results:**

10,779 participants were included. Before and after PSM, gallstone group individuals had higher DII scores than non-gallstone group individuals (*p* < 0.05). Matched logistic regression analysis showed that DII scores were positively correlated with gallstone risk (adjusted OR = 1.14, 95% CI 1.01, 1.29). The stratified analysis showed that this association was stronger in overweight or obese people (adjusted OR = 1.18, 95% CI 1.03, 1.34). RCS analysis suggested that DII and gallstones showed a “J”-shaped non-linear dose–response relationship (*p* non-linear <0.001).

**Conclusion:**

Higher DII score is positively associated with the risk of gallstones, particularly in overweight or obese population, and this relationship is a “J”-shaped non-linear relationship. These results further support that avoiding or reducing a pro-inflammatory diet can be an intervention strategy for gallstone management, particularly in the overweight or obese population.

## 1 Introduction

Gallstones and complications are common reasons for gastroenterology hospitalization (1). The prevalence of gallstones varies by income region, with 20% reported in Europe and other developed countries, 5 to 20% in Asia, and 3 to 5% in Africa (2, 3). The incidence of gallstones also increases in children due to the obesity epidemic (4). Studies have shown that gallstones increase the risk of diabetes, tumors, and all-cause mortality, which will cause suffering and severe financial burden of disease to patients (5). About 75% of adult patients are asymptomatic (6) but rely more heavily on surgical treatment if symptoms or complications develop. Approximately 750,000 cholecystectomies are performed annually in the United States (7). Although minimally invasive surgery is effective in the treatment of gallstones and has a low mortality rate, the incidence of post-cholecystectomy syndromes such as abdominal pain, jaundice, and dyspepsia was as high as 40% in one study, and the onset time ranged from 2 days to 25 years (8, 9), which still greatly affected patients’ quality of life. Therefore, exploring the primary prevention of gallstones (changes in diet and lifestyle) may be beneficial in reducing the prevalence and health care costs.

Over 80% of gallstones are composed of cholesterol (10) and are affected by various factors, including age, sex, pregnancy, obesity, sedentary lifestyle, diet, and inflammatory response (11, 12). Among all risk factors, inflammation has received much attention. Studies have revealed that inflammatory biomarkers such as IL-6, IL-8, TNF-α and CRP are associated with an increased risk of gallstones. On the contrary, IL-4 is associated with a decreased risk of gallstones (13, 14). In recent years, dietary inflammation has been introduced, and specific dietary components are thought to be possible modifiers of chronic inflammation. The Western diet has been reported to significantly increase systemic inflammatory biomarkers such as CRP, IL-6, and IL-10, associated with an increased risk of gallstones (15, 16). In contrast, the Mediterranean diet has reduced the aforementioned inflammatory biomarkers and is associated with a lower risk of gallstones (17, 18).

Dietary patterns can be characterized as pro-inflammatory or anti-inflammatory (19). The Dietary Inflammatory Index (DII), developed by Cavicchia et al. (20) and updated by Chiappa et al. (21), is an effective tool for measuring the inflammatory potential of individual dietary intake. Numerous studies have indicated that high DII is associated with the risk or prognosis of multiple diseases, including cardiovascular disease (22), diabetes (23), cancer (24) and so on. Although the relationship between diet and gallstones has been extensively studied, it has focused on specific nutrients, dietary patterns, and eating habits. To our knowledge, relatively few studies have investigated the underlying inflammatory diet patterns and the development of gallstones.

Understanding the relationship between an inflammatory diet and the risk of gallstones is vital for adapting intervention strategies related to dietary modifications to reduce inflammation. Therefore, in this study, we used an extensive US population-based survey database, the National Health and Nutrition Examination Survey (NHANES) 2003–2020, to assess the association between gallstones and DII and to explore the dose–response relationship between the two.

## 2 Materials and methods

### 2.1 Study design and population

The NHANES is one of a series of health-related programs conducted by the National Center for Health Statistics (NCHS) to provide information on the health and nutritional status of adults and children in the United States. It uses a stratified multistage sampling design to collect samples from a nationally representative sample of US civilians, and the details of the survey design and methodology can be found on the NHANES website.^1^ NHANES has collected data continuously since 1999 and publishes them publicly on a biennial cycle, using sample weights for all analyses and taking into account the complex design of the survey when calculating standard errors. NCHS and the Centers for Disease Control and Prevention (CDC) Institutional Review Board approved the NHANES protocol, and all participants provided written informed consent (25).

**Figure 1:**
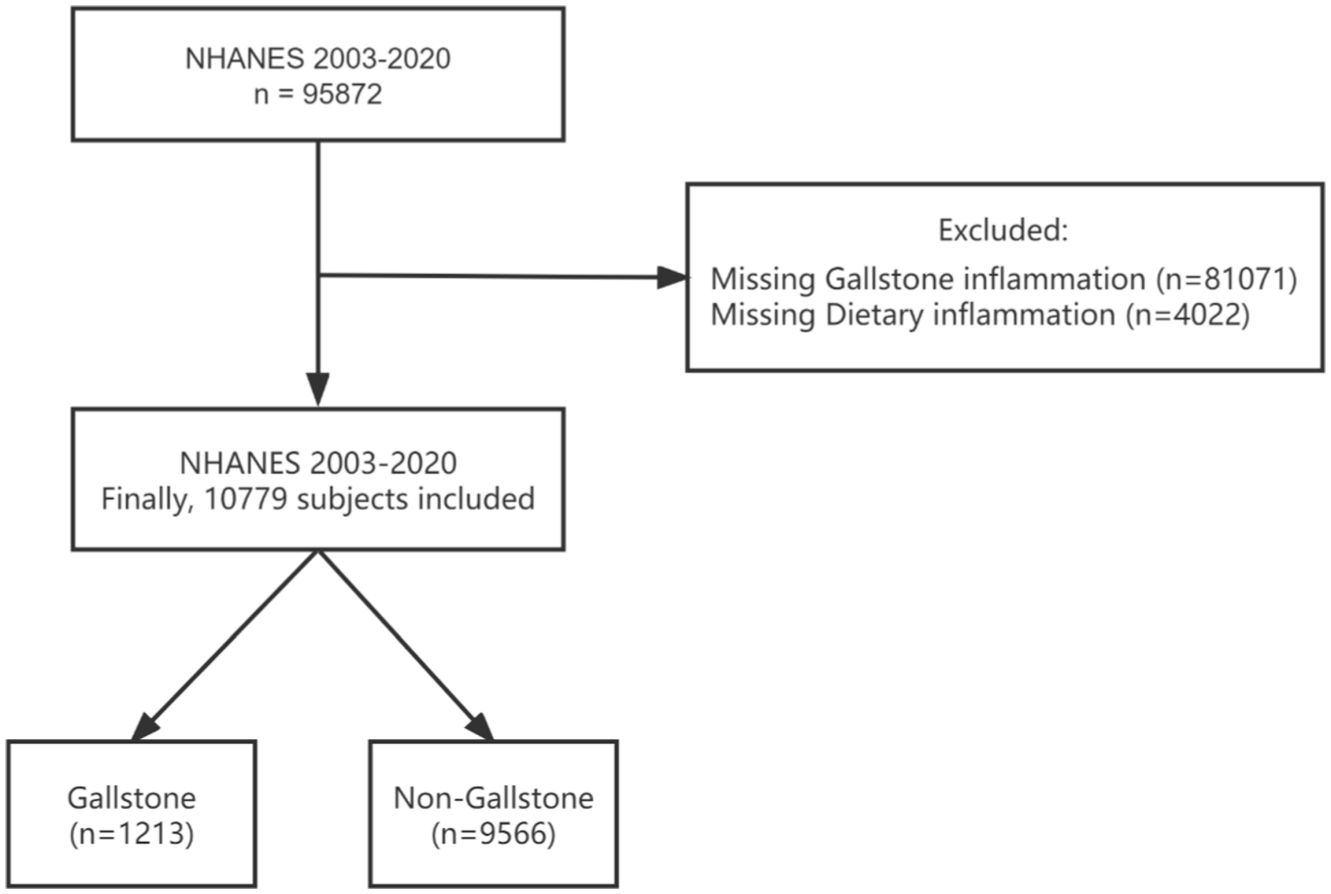
Flowchart of the sample selection from NHANES 2003–2020. NHANES, National Health and Nutrition Examination Survey.

This study combined the NHANES database for nine consecutive periods from 2003 to 2020. A total of 95,872 people participated in the survey. The participants younger than 20 years of age with missing DII data and gallstone data were excluded from our study. Referring to previous literature (26, 27), gallstones were determined based on the Medical Condition Questionnaire data. When participants were asked, “Have you ever been told by a doctor or professional that you have gallstones?” If the answer was “yes,” the participant was considered to have gallstones. The accuracy of self-reported gallstones has been reported elsewhere. Tsai et al. (28) have confirmed the validity of self-reported stones in the Health Professionals Follow-Up Study by analysing a random sample of 441 medical records of participants who reported a cholecystectomy or gallstones were reviewed, and of these, the diagnosis was confirmed in all except for 5 participants (99%). The present study ultimately included 10,779 participants (Figure 1).

### 2.2 Dietary inflammation index calculation

In this study, dietary intake information was assessed using the 24-h dietary recall interviews, and all dietary data were validated by the Nutrition Methodology Working Group (29). DII was calculated according to the method published by Chiappa et al. (21). They summarized the inflammatory effect scores for 45 kinds of nutrients and estimated the global means with standard deviations for each nutrient by combining results from 11 populations worldwide. The DII score could be used fully or partially of these 45 kinds of nutrients. The NHANES project includes 27 of the 45 dietary nutrients listed above (30). Consequently, the following nutrients were used to calculate the DII score: caffeine, alcohol, beta-carotene, total sugar, cholesterol, energy, total fat, dietary fiber, lycopene, folic acid, selenium, iron, magnesium, total polyunsaturated fatty acids, total monounsaturated fatty acids, total saturated fatty acids, protein, riboflavin, thiamin, vitamin A/C/D/E/B6/B12, zinc and carbohydrate.

Here is a brief description of the calculation method. First, the average daily intake of dietary nutrients in individual dietary components was obtained using the 24-h dietary recall interviews. Second, the Z score of each dietary ingredient was calculated by comparing the individual dietary ingredient intake with the global average daily intake and standard deviation, which took from the paper (21). Third, to minimize bias, all Z scores were converted to percentiles. Each percentile score was doubled, and then ‘1’ was subtracted to achieve a symmetrical distribution with values centered on 0 (null) and bounded between-1 (maximally anti-inflammatory) and + 1 (maximally pro-inflammatory). Fourth, the values obtained from centralization were multiplied by the respective overall food parameter-specific inflammatory effect scores provided in the literature to calculate the DII score for that dietary component of the individual (21). Finally, the DII score of all dietary components of the individual was added to obtain the overall DII score. The resulting DII score >0, = 0 and <0 represent pro-inflammatory diet, non-inflammatory diet and anti-inflammatory diet, respectively (21, 31).

### 2.3 Covariates

Covariates in this study included sex, age, race, education level, household income to poverty income ratio (PIR), body mass index (BMI), smoking, alcohol, sleep, physical activity, marital status, diabetes, hypertension, and high cholesterol. PIR was divided into: low (PIR <1.3), medium (PIR1.3–3.5) and high (PIR >3.5) (32). Smoking status was classified into: never (<100 cigarettes), former (≥ 100 cigarettes and smoke not at all now), current (≥ 100 cigarettes and smoke some days or every day) (33). Participants were defined as drinking if they had at least 12 drinks a year. Physical activity was a dichotomous variable, with yes representing moderate or vigorous intensity sports, fitness, or recreational activities in a typical week. Based on previous research (28, 34, 35), participants were considered to have diabetes if one of the following was true: (1) “Doctor told they have diabetes”; (2) “Taking diabetic pills now.” The determination of hypertension and high cholesterol was similar to this.

### 2.4 Statistical analysis

Data were processed using RStudio (version 4.2.2) and Excel (version 2016). According to the analysis recommendations on the website of the NHANES, survey weights were constructed for all survey years. Normally distributed continuous data were described by mean ± SD, categorical data were characterized by frequency (*n*) and percentage (%). Comparisons between groups were made using the *χ*^2^ test and the Wilcoxon rank sum test. To reduce the influence of confounding factors on study results, we used the Matchets package of RStudio to perform PSM on confounding factors such as gender, age, race, PIR, BMI, smoking, physical activity, and marital status. The matching ratio was 1:1, the scale value was 0.02, and the closest matching method was adopted. Based on the matched data, four binary logistic regression models were applied to analyze the relationship between DII and gallstones. Finally, a restricted cubic spline (RCS) curve model was used to plot the dose–response relationship between DII and gallstones. Four nodes, P5, P35, P65, and P95, were selected for the DII and the DII corresponding to P50 was used as the reference value. In the subgroup analysis, the data were stratified according to BMI into overweight or obese (BMI ≥ 25 kg/m^2^) and non-obese (BMI< 25 kg/m^2^) to further explore the effects of DII on different people. *p <* 0.05 was considered statistically significant.

## 3 Results

### 3.1 Characteristics of the study population before and after PSM

Of the 10,779 participants, the age range was 20–80 years, and the mean was 48 ± 17 years. About 52% of the subjects were females, 63% were non-Hispanic white (Table 1). The prevalence of gallstones was 11% (1,213/9566), and the average of DII was 1.25 ± 1.80. Before PSM, statistically significant differences were found between the gallstone and non-gallstone groups on variables such as gender, age, PIR, marital status, BMI, smoking, physical activity, diabetes, hypertension, and high cholesterol (*p <* 0.05). Compared to the non-gallstone group, the gallstone group had higher age and BMI, lower income, less physical activity, more women than men. They were likelier to be smoking with co-occurring diabetes, hypertension and high cholesterol. After 1:1 caliper matching treatment with PSM, 1205 pairs of gallstone and non-gallstone groups were successfully matched. After PSM, there was no statistical significance in the comparison of other covariates between the two groups except PIR. It is worth noting that DII in the gallstone group was significantly higher than that in the non-gallstone group before and after PSM. The difference was statistically significant (*p =* 0.017 and *p =* 0.006).

**Table 1:** Comparison of baseline features before and after PSM.^1^

	Before PSM			After PSM		
Characteristic	Non-gallstone, *N* = 9,566 (89%)^1^	Gallstone, *N* = 1,213 (11%)^1^	*p* value^2^	Non-gallstone, *N* = 1,205 (52%)^1^	Gallstone, *N* = 1,205 (48%)^1^	*p* value^2^
Sex			**<0.001**			0.4
Male	4,795 (50%)	351 (27%)		348 (31%)	351 (27%)	
Female	4,771 (50%)	862 (73%)		857 (69%)	854 (73%)	
Age (years)			**<0.001**			0.2
20–39	2,892 (37%)	170 (16%)		177 (14%)	170 (16%)	
40–50	1,511 (15%)	170 (17%)		173 (12%)	170 (17%)	
50+	5,163 (48%)	873 (66%)		855 (73%)	865 (66%)	
Race			0.3			0.055
Non-Hispanic white	3,356 (62%)	543 (66%)		537 (75%)	539 (66%)	
Non-Hispanic black	2,655 (12%)	245 (7.7%)		254 (6.8%)	245 (7.7%)	
Other/multiracial	1,546 (10.0%)	151 (12%)		176 (6.6%)	148 (12%)	
Mexican American	1,107 (8.7%)	146 (8.2%)		126 (6.5%)	145 (8.3%)	
Other Hispanic	902 (6.9%)	128 (6.0%)		112 (4.9%)	128 (6.0%)	
Education level			0.4			0.7
<High school	1,608 (9.7%)	189 (8.7%)		174 (7.6%)	187 (8.6%)	
High school	2,241 (27%)	304 (31%)		285 (29%)	303 (31%)	
>High school	5,717 (64%)	720 (60%)		746 (63%)	715 (60%)	
PIR			**0.003**			**0.048**
Low	2,679 (20%)	319 (23%)		323 (18%)	317 (23%)	
Middle	3,756 (35%)	537 (43%)		546 (37%)	531 (43%)	
High	3,131 (46%)	357 (34%)		336 (45%)	357 (34%)	
BMI (kg/m^2^)			**<0.001**			0.4
<18.5	141 (1.5%)	7 (0.3%)		5 (0.1%)	7 (0.3%)	
18.5 to<25	2,367 (26%)	137 (12%)		140 (12%)	137 (12%)	
25 to<30	3,025 (30%)	323 (27%)		356 (35%)	323 (27%)	
≥30	4,033 (42%)	746 (61%)		704 (53%)	738 (61%)	
Smoking status			**0.001**			0.7
Current smoker	1,741 (16%)	191 (16%)		185 (13%)	191 (16%)	
Former smoker	2,253 (24%)	386 (32%)		354 (31%)	380 (32%)	
Never smoker	5,572 (60%)	636 (52%)		666 (56%)	634 (52%)	
Alcohol			0.8			>0.9
No	2,862 (30%)	355 (31%)		349 (32%)	352 (31%)	
Yes	6,704 (70%)	858 (69%)		856 (68%)	853 (69%)	
Sleep(h)			0.8			0.9
≤6	1,875 (18%)	238 (17%)		195 (18%)	234 (17%)	
6 ~ 9	6,646 (72%)	831 (72%)		883 (73%)	827 (72%)	
>9	1,045 (9.6%)	144 (11%)		127 (9.4%)	144 (11%)	
Physical activity			**0.009**			0.2
No	4,893 (43%)	739 (53%)		690 (48%)	732 (53%)	
Yes	4,673 (57%)	474 (47%)		515 (52%)	473 (47%)	
Marital status			**0.001**			0.7
Married	5,753 (63%)	739 (57%)		699 (61%)	735 (57%)	
Divorce/separate/widowed	2,154 (17%)	325 (29%)		333 (27%)	323 (29%)	
Single	1,659 (20%)	149 (14%)		173 (12%)	147 (14%)	
Diabetes			**<0.001**			0.4
No	7,857 (86%)	825 (74%)		935 (78%)	823 (74%)	
Yes	1,709 (14%)	388 (26%)		270 (22%)	382 (26%)	
Hypertension			**<0.001**			0.7
No	6,016 (69%)	552 (50%)		602 (49%)	551 (51%)	
Yes	3,550 (31%)	661 (50%)		603 (51%)	654 (49%)	
High cholesterol			**<0.001**			0.2
No	6,257 (69%)	622 (51%)		656 (57%)	620 (51%)	
Yes	3,309 (31%)	591 (49%)		549 (43%)	585 (49%)	
DII	1.20 ± 1.80	1.55 ± 1.76	**0.017**	0.95 ± 1.68	1.55 ± 1.76	**0.006**

Bold values represent *p* values <0.05. PIR, Poverty Income Ratio; BMI, Body Mass Index; DII, Dietary Inflammatory Index.

^1^Median (IQR) for continuous; *n* (%) for categorical.

^2^Chi-squared test with Rao & Scott’s second-order correction; Wilcoxon rank-sum test for complex survey samples.

### 3.2 Correlation analysis of DII and gallstones in different populations after PSM

**Figure 2:**
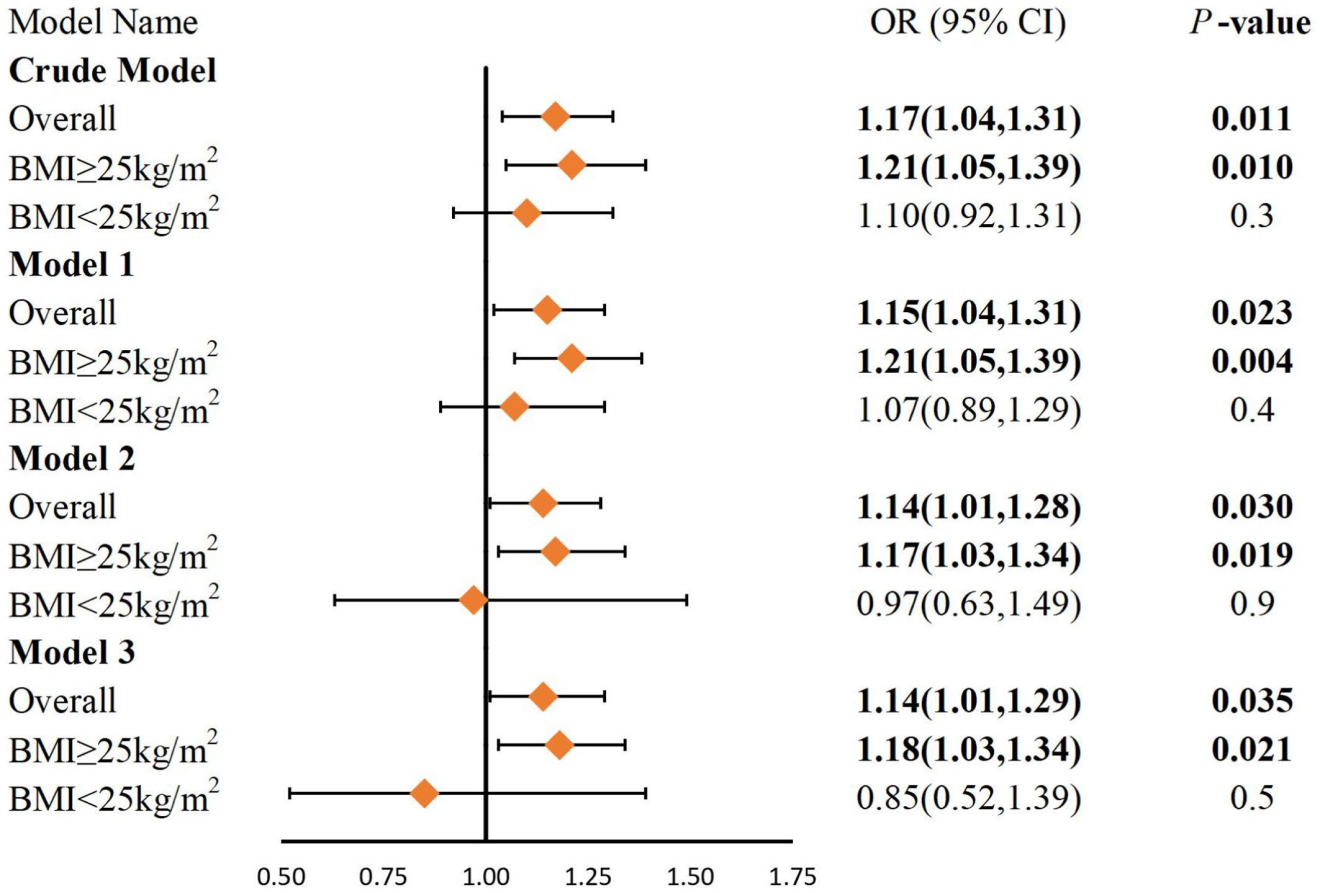
Logistic regression analysis of DII and gallstones after PSM. BMI, Body Mass Index. Crude model: No covariates were adjusted. Model 1: Adjustments made for age, sex, race, education level, and PIR. Model 2: Adjustments same as that in model 1 plus BMI, physical activity, smoking status, alcohol, sleep and marital status. Model 3: Adjustments same as that in model 2 plus diabetes, hypertension and high cholesterol.

As shown in Figure 2, we build four multivariate logistic regression models. In all models, we observed a positive correlation between DII and gallstones. After adjusting for all confounders, each unit increase in DII increased the odds of having gallstone by 14% (OR 1.14, 95% CI 1.01, 1.29, *p* = 0.035). The results of the subgroup analysis showed that this relationship was more significant in overweight or obese people (OR 1.18, 95% CI 1.03, 1.39, *p* = 0.021). However, no meaningful results were observed in the non-obese population.

### 3.3 RCS analysis of DII and gallstones in different populations after PSM

**Figure 3:**
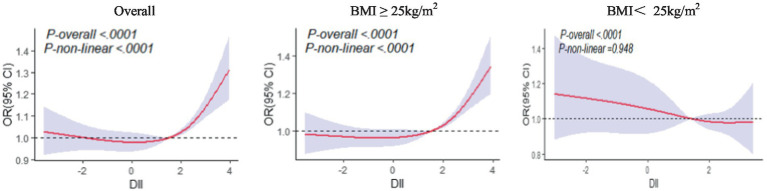
Restricted cubic spline models for the relationship between DII and the risk of gallstones in different populations after PSM. DII, Dietary Inflammatory Index. Adjusted OR and 95% CI are indicated by red lines and shades of blue. The model adjusted for age, sex, race, education level, PIR, BMI, physical activity, smoking status, alcohol, sleep, marital status, diabetes, hypertension and high cholesterol.

To more clearly elucidate the relationship between DII and the risk of gallstones, we performed RCS analyses on the total population and the population stratified by BMI after PSM. After unadjusted models and adjusted for confounding factors including age, sex, race, education level, PIR, BMI, physical activity, smoking status, alcohol, sleep, marital status, diabetes, hypertension and high cholesterol, the RCS showed (Figure 3) a “J”-shaped non-linear dose–response relationship between DII and gallstone risk in the total population as well as in the overweight or obese population (*p* non-linear < 0.001). In the total population and overweight or obese population, the risk of gallstone decreased with increasing DII when the DII score < 0 and increased with increasing DII when the DII score> 0. However, in the non-obese population, the results were not significant (*p* non-linear > 0.05).

## 4 Discussion

This large retrospective study explored the relationship between potential dietary inflammation and gallstone risk based on the dietary inflammation index, and obtained some meaningful findings. Firstly, in this study, age, female gender, overweight and obesity, smoking, low income, lack of physical activity, diabetes, and high blood pressure were all risk factors for gallstones. Our study is consistent with some previous research in this field (36, 37). Some studies have suggested a link between alcohol consumption and gallstones (38), however, this study did not get the same finding. This may be due to the limitations of the data dichotomy on alcohol consumption in this database, suggesting that it is necessary to subdivide alcohol consumption when data are collected. Secondly, the most obvious result of this paper was that both before and after PSM, DII was significantly higher in the gallstone group than in the non-gallstone group, and DII was positively directly associated with gallstone risk. Further, according to the results of the RCS analysis, there was a “J” shaped non-linear relationship between DII and gallstones in the total population. It is revealed DII levels is a risk factor for gallstones. The stratified analysis found that DII was a significant risk factor for overweight or obese people, and the non-linear association persisted in the DII and overweight or obese population.

In this study, our results based on 10,779 adults from NHANES showed gallstone risk increases with increasing DII, and there was a “J”-shaped non-linear relationship. It is worth emphasizing that the risk of gallstones decreases with increasing DII when DII < 0 and increases with increasing DII when DII > 0. This means that the risk of gallstones decreases with anti-inflammatory diet intake and increases with increased pro-inflammatory diet intake. However, a study by Sadri et al. (36) indicated that DII was associated with a reduced incidence of gallstones. The reasons are as follows: First, the two studies’ demographic characteristics, sample size and prevalence were different. In this study, NHANES data from 2003 to 2020 pooled 10,779 participants, all of whom were US citizens, and the prevalence of gallstones was 11%. Their study used Persian cohort data and included 3,626 participants with a gallstone prevalence of 4.77%. Previous studies have shown regional differences in the prevalence of gallstones. Approximately 10 to 20 percent of US adults have gallstones (39), whereas in Iran are less than 1 percent (40). Secondly, the DII range and its composition are different. In this study, 27 food parameters were used to calculate DII. Before matching, the DII range was (−4.96 ~ 4.70), and about 73% of the participants’ DII were pro-inflammatory. After matching, the DII range was (−4.53 ~ 4.60), and about 75% of the participants’ DII were pro-inflammatory. Their study used 34 food parameters to calculate DII, and the final DII had a maximum value of 1.43, and about 30% of participants had pro-inflammatory DII. Although the NHANES project contained only 27 dietary nutrients, previous research has stated that the predictive power of DII remains stable even with <30 dietary nutrients (41). Finally, the analytical methods used, the covariates included, and the confounding factors adjusted for differences between the two studies may also impact the results. Therefore, future clinical studies are needed to elucidate the relationship between DII and gallstone risk.

This study performed a subgroup analysis by BMI stratification to explore the effects of DII in different populations. We found a significant impact of DII on the overweight or obese population. RCS analysis further demonstrated a non-linear association between DII and the overweight or obese population. This finding is similar to the results mentioned earlier. It suggests that consumption of a pro-inflammatory diet may be one of the roles in the risk of gallstones in overweight or obese individuals. Unfortunately, no meaningful results were observed in the non-obese population. The reasons may be: (1) There is a correlation between obesity and gallstones (42). Stender et al. (43) revealed that BMI was a risk factor for gallstones, more pronounced in women. Symptomatic gallstone risk increased by 7 to 8 percent for each unit increase in BMI. A Mendelian randomized study of obesity, type 2 diabetes, lifestyle factors, and risk of gallstones by Yuan et al. (44) indicated a positive association between obesity and gallstones (OR 1.63, 95% CI 1.49, 1.79). The pathophysiological mechanisms underlying the increased risk of gallstones in obese individuals are multifactorial, and mainly consist of altered metabolic factors, supersaturated bile secretion by the liver, dyslipidemia, low intestinal and gallbladder motility, gallbladder stasis, reduced bile acid secretion, cholesterol crystallization and precipitation, and supersaturation of gallbladder bile, among other mechanisms (45). (2) Obesity is a significant risk factor for metabolic diseases. Compared to non-obese populations, obese people often have comorbidities such as diabetes mellitus, non-alcoholic fatty liver disease, cardiovascular disease and metabolic syndrome (46), all of which have been shown to be associated with an increased risk of gallstones (47–51). (3) Several studies have evaluated the relationship between DII and overweight or obesity. A prospective cohort study in Spain proved that DII was associated with weight gain and a higher risk of being overweight or obese (52). A cross-sectional survey by Ruiz-Canela et al. (53) also revealed that DII was positively associated with higher BMI. It is worth highlighting in particular that although this study did not find meaningful results in non-obese people, this does not mean that non-obese people are free to consume a high DII diet. Although the risk of gallstones is relatively low in non-obese people, the formation of gallstones is not exclusively associated with obesity. Non-obese people may also have an increased risk of gallstones due to factors such as genetics, dietary habits, lack of exercise, increasing age, rapid weight loss, pregnancy, or the use of some medications (2, 11, 54). Therefore, non-obese people still need to be aware of other potential risk factors.

This study has several strengths. First, this study utilized PSM to balance the confounding bias between the gallstone and non-gallstones group, ensuring good comparability and reliability of conclusions between the two groups. Second, this study adopted RCS analysis to further demonstrate the nonlinear associations between DII and gallstones. However, our study still has some limitations. First, the DII used in this study was collected through 24-h dietary recall, and the disease data used were based on self-reported disease histories with possible recall bias. Second, we cannot distinguish between cholesterol stones and bile pigment stones, which have different etiologies. Bile pigment stones may not be affected by the covariates included in this study, and further classification studies can be carried out in the future. Third, although we included many covariables in the analytical model, we cannot completely rule out the influence of confounding factors that are not included or are unknown. Moreover, due to the lack of a large number of inflammatory biomarker data in this study, it was not included in the analysis. However, future studies need to consider the impact of inflammatory biomarkers on the findings. Finally, because the data from our study were from a cross-sectional survey and the timing of DII and gallstones could not be determined, causal inference cannot be made and further prospective studies are needed to confirm these findings in the future.

## 5 Conclusion

This study is a large cross-sectional study based on the NHANES database to assess the relationship between DII and gallstone risk. We found that gallstone risk increases with increasing DII, and there was a “J”-shaped non-linear relationship between DII and gallstones, and this relationship was more pronounced in those who were overweight or obese. These results further support that avoiding or reducing a pro-inflammatory diet can be an intervention strategy for gallstone management, particularly in the overweight or obese population.

## Data availability statement

Publicly available datasets were analyzed in this study. This data can be found here: https://www.cdc.gov/nchs/nhanes/index.htm.

## Ethics statement

The studies involving humans were approved by NCHS and the Centers for Disease Control and Prevention (CDC) Institutional Review Board approved the NHANES protocol. The studies were conducted in accordance with the local legislation and institutional requirements. The participants provided their written informed consent to participate in this study. Written informed consent was obtained from the individual(s) for the publication of any potentially identifiable images or data included in this article.

## Author contributions

YL: Resources, Writing – original draft, Visualization, Validation, Software, Methodology, Investigation, Formal analysis, Data curation, Conceptualization. XG: Writing – review & editing, Funding acquisition, Formal analysis, Conceptualization. MX: Writing – review & editing, Funding acquisition, Formal analysis. FY: Writing – review & editing, Supervision, Methodology. XZ: Methodology, Supervision, Writing – review & editing. GQ: Methodology, Supervision, Writing – review & editing. CX: Writing – review & editing, Supervision. TJ: Writing – review & editing, Supervision, Funding acquisition.

## Footnotes

^1^ https://www.cdc.gov/nchs/nhanes/index.htm
